# The dark side of ZNF217, a key regulator of tumorigenesis with powerful biomarker value

**DOI:** 10.18632/oncotarget.5893

**Published:** 2015-09-29

**Authors:** Pascale A. Cohen, Caterina F. Donini, Nhan T. Nguyen, Hubert Lincet, Julie A. Vendrell

**Affiliations:** ^1^ ISPB, Faculté de Pharmacie, Lyon, France; ^2^ Université Lyon 1, Lyon, France; ^3^ INSERM U1052, CNRS UMR5286, Centre de Recherche en Cancérologie de Lyon, Lyon, France

**Keywords:** ZNF217, oncogene, biomarker, hallmarks of cancer, carcinogenesis

## Abstract

The recently described oncogene *ZNF217* belongs to a chromosomal region that is frequently amplified in human cancers. Recent findings have revealed that alternative mechanisms such as epigenetic regulation also govern the expression of the encoded ZNF217 protein. Newly discovered molecular functions of ZNF217 indicate that it orchestrates complex intracellular circuits as a new key regulator of tumorigenesis. In this review, we focus on recent research on ZNF217-driven molecular functions in human cancers, revisiting major hallmarks of cancer and highlighting the downstream molecular targets and signaling pathways of ZNF217. We also discuss the exciting translational medicine investigating ZNF217 expression levels as a new powerful biomarker, and ZNF217 as a candidate target for future anti-cancer therapies.

## INTRODUCTION

During tumor progression, normal cells evolve progressively to a neoplastic state through the successive acquisition of hallmark capabilities. The hallmarks of cancer - distinctive and complementary capabilities that enable tumor growth and metastatic dissemination - continue to provide a solid foundation for understanding the biology of cancer and have been remarkably reviewed by Hanahan and Weinberg [1, 2]. The numerous signaling molecules orchestrating tumorigenesis operate through elaborate integrated circuits governing intracellular signaling networks. In this context, discovering new master regulators and their functional roles, is of utmost interest.

The zinc-finger protein 217 (ZNF217) is an oncogenic protein that plays deleterious functions in various human cancers. The *ZNF217* gene is located at the 20q13 chromosomal region [3], which is frequently amplified in human tumors [4]. This region also contains several oncogenes thought to confer selective advantages to cancer cells. Increased copy numbers of *ZNF217* have been reported in various tumors (frequency is variable among tumors) [5-7] and linked to poor outcome in some studies [8-11].

The ZNF217 protein is a member of the Kruppel-like family of transcription factors and contains 8 predicted C2H2 zinc finger motifs and a proline-rich region [3]. It binds to specific DNA sequences to regulate target gene expression [12, 13], is a component of a human histone deacetylase complex (CoREST-HDAC) [12, 14, 15], and is found in complexes with the transcriptional co-repressor C-terminal binding protein (CtBP), the histone demethylases LSD1 and KDM5B/JARID1B/PLU-1, and the methyltransferases G9a and EZH2 [8, 12, 14-18]. ZNF217 was first described to mainly act as a transcriptional repressor complex, as reviewed previously [8], however additional research has shown that it positively regulates the expression of specific target genes [13, 15, 19, 20], making it a complex and double-faceted transcriptional regulator.

An increasing body of research indicates that ZNF217 interferes with several intracellular signaling networks for reprogramming cancer cells. Evidence also suggests that ZNF217 expression levels are not systematically associated with the *ZNF217* gene amplification status and that other events, such as the action of miRNAs [21-23] and promoter methylation [24-26], also impact *ZNF217* expression. It is therefore important to decipher the complex molecular events that this protein may orchestrate, aside from *ZNF217* genomic amplification. In this review, we revisit ZNF217-driven molecular functions in human cancers through the major hallmarks of cancer [1, 2]: sustained proliferative signals, evasion from growth suppressors, replicative immortality, resistance to cell death, cancer stem cell enrichment, and activation of invasion and metastasis. We will focus on the molecular targets and signaling pathways associated with or activated by ZNF217 as well as recently identified ZNF217 protein partners. This review will also describe the evolving landscape of translational medicine with regards to assessing ZNF217 expression levels (mRNA or protein) as a new prognostic or predictive biomarker for anti-cancer therapies. Finally, we will discuss the emerging therapeutic strategies to counteract ZNF217 or ZNF217-driven deleterious effects in cancer.

### Sustained proliferative signals and disruption of negative-feedback mechanisms that attenuate proliferative signaling

**Figure 1:**
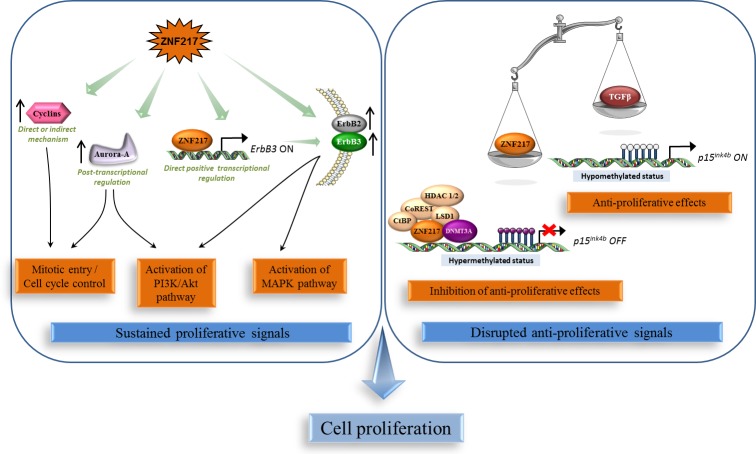
ZNF217 sustains chronic cell proliferation Left panel: ZNF217 induces deregulation of proliferative signals via the over-expression and/or activation of key players such as cyclins, Aurora-A, the oncogenic unit ErbB2-ErbB3 and the survival PI3K/Akt and MAPK pathways. Right panel: ZNF217 also disrupts anti-proliferative signals and impairs the anti-proliferative TGF-β-dependent program through inhibition of the recruitment of cofactors involved in the active demethylation of the *p15^ink4b^* gene.

ZNF217 has been depicted to impact one of the most fundamental traits of cancer cells involving their ability to sustain chronic proliferation via the deregulation of signals that allow progression through the cell cycle, cell growth and disruption of anti-proliferative signaling. Indeed, *in vitro* overexpression of ZNF217 promoted cell proliferation in ovarian [27] and breast cancer cells [28], whereas silencing of *ZNF217* reduced it in prostate [23], colorectal [21], ovarian [7, 29] and breast [28] cancer cells. *In vivo*, constitutive expression of ZNF217 in breast or ovarian cancer cells stimulated the growth and the rate of tumor formation [20, 27, 28, 30]. The rapid proliferation of ZNF217-overexpressing cells was correlated with a significant increase in S phase population [27], aberrant expression of several cyclins and, by a post-transcriptional mechanism, with aberrant elevated expression of Aurora-A (Aurora kinase A/AURKA/STK15) [28] (Figure 1).

The *ErbB3* gene was identified as a direct target for the ZNF217 transcription factor and was the first gene shown to be positively regulated by recruitment of ZNF217 to its promoter [19]. *ZNF217* and *ErbB3* expression levels showed significant correlation in human breast tumors [19], and ectopic ZNF217 expression induced ErbB3 protein overexpression in normal human mammary epithelial cells (HMECs) and in breast cancer cell lines (paired with increased ErbB2 expression) [19, 20]. ZNF217 also sensitized breast cancer cells to heregulin, the growth factor ligand for ErbB3 [30]. The ErbB2-ErbB3 heterodimer functioning as an “oncogenic unit”, the global impact of ZNF217 on ErbB receptor expression maybe one possible mechanism by which ZNF217 augments the PI3K/Akt, and possibly the MAPK survival pathways [19, 30] (Figure 1).

The p15^ink4b^ tumor suppressor, which inhibits cell-cycle progression at the G1/S transition [31], has been identified as a direct target of the ZNF217/CoREST transcriptional complex [15]. This complex induced transcriptional silencing of the *p15^ink4b^* gene through the co-recruitment of the DNMT3A DNA methyltransferase and promoter hypermethylation [14]. Importantly, ZNF217 overexpression was sufficient to impair the anti-proliferative TGF-β-dependent program through inhibition of the recruitment of cofactors involved in the active demethylation of the *p15^ink4b^* gene [14]. Corruption of the TGF-β pathway is known to promote malignancy at an early stage by stimulating evasion of cancer cells from TGF-β anti-proliferative effects [32, 33]. Thus alteration of DNA methylation status of target genes such as *p15^ink4b^* is part of the ZNF217-dependent disruption of anti-proliferative signals at the early stage of tumorigenesis (Figure 1).

### Enabling replicative immortality

The first study suggesting a link between ZNF217 and senescence demonstrated that transduction of finite life span HMECs with *ZNF217* gave rise to immortalized cells with increased telomerase activity [34]. These cells also exhibited an increased resistance to TGF-β-induced anti-proliferative action while alterations in p53 and/or pRB did not seem to contribute [34]. A second study showed that *ZNF217* transduction into SV40 Tag/tag expressing, p53/pRB-deficient, human ovarian surface epithelial cells (named IOSE cells) promoted neoplastic progression associated with telomerase activity, anchorage independence, genomic changes and aberrant gene-expression levels similar to those observed in ovarian carcinomas [35]. However, the immortalization driven by ZNF217 could not be obtained in ovarian surface epithelial (OSE) cells which possess functional p53 and pRB. The oncogenic translation elongation factor eEF1A2 [36] is overexpressed in *ZNF217*-transduced IOSE cells [35], and transduction of *eEF1A2* in the same cells was sufficient to induce neoplastic progression [37]. Finally, *eEF1A2* silencing reversed resistance to apoptosis induced by ZNF217 overexpression, thus highlighting eEF1A2 as contributing towards the ZNF217-induced neoplastic properties of these precursor cells of ovarian carcinomas [37]. The *eEF1A2* gene is, like *ZNF217,* located on chromosome 20q13. As this locus is not necessarily amplified in the ZNF217-immortalized IOSE cells [35] and as interaction between ZNF217 protein and *eEF1A2* gene regulatory regions appears negligible [13], the authors suggested that ZNF217-driven *eEF1A2* transcription likely occurs through an indirect mechanism, perhaps involving the ZNF217-induced inhibition of negative regulator(s) of *eEF1A2* expression [37]. Altogether, these studies demonstrate that *ZNF217* is a new oncogene that drives neoplastic progression in breast and ovarian cancers, and may act interdependently with eEF1A2. As HMECs lack functional p16^INK4a^ and IOSE cells lack functional p53 and pRB, defects in these principal regulators of proliferation and senescence might be important contributing factors in ZNF217-mediated immortalization and/or neoplastic progression.

Both immortalized *ZNF217*-transduced HMECs and IOSE cells display stabilization of telomere length and increased telomerase activity [34, 35], both associated with senescence bypassing [38, 39]. ZNF217 attenuated apoptotic signals resulting from functionally compromised telomeres [40] and ZNF217-immortalized HMECs displayed increased expression of telomere repeat binding factor 2 (TRF2), as a likely consequence of post-transcriptional regulation [41]. As artificially overexpressed TRF2, is involved in safeguarding telomere structures [42] and reported to delay senescence [43], one could reasonably postulate that TRF2 mediates, at least in part, ZNF217-driven immortalization. Telomerase expression and activity can be enhanced through the PI3K/Akt pathway [44] and a link between ZNF217 and activation of the PI3K/Akt pathway has been repeatedly shown [19, 30, 40]. Future work is necessary to decipher whether ZNF217 is involved, directly or indirectly, in any post-transcriptional events driving TRF2 overexpression and in any Akt-associated activation of the telomerase. Altogether, ZNF217 has been identified as a new oncogene acting at early stages of carcinogenesis, and capable of bypassing senescence and promoting immortalization.

### Resisting cell death

ZNF217 has been shown to interfere with the apoptotic pathway at early stages of tumor progression, by impairing apoptotic signals resulting from dysfunctional telomeres [40], and in later stages, by conferring resistance to chemotherapy [28, 40]. Doxorubicin is a chemotherapeutic agent that inhibits topoisomerase II and induces double-strand DNA breaks resulting in ATM-dependent p53-mediated apoptosis. Taxanes such as paclitaxel are microtubule-stabilizing agents that cause cell cycle arrest and apoptosis. Ectopic expression of ZNF217 conferred both doxorubicin and paclitaxel resistance in breast cancer cells, and conversely, *ZNF217* silencing increased doxorubicin or paclitaxel sensitivity [28, 40]. This ZNF217-driven drug resistance was not achieved through the ABCB1/PgP transporter [28], but rather by ZNF217 interfering with the apoptotic signals induced by the two drugs [28, 40]. ZNF217-driven paclitaxel or doxorubicin resistance in MDA-MB-231 cells is most likely p53-independent, given that these cells possess a nonfunctional mutated p53 [28], while activation of a p53-dependent pathway involved in ZNF217-mediated doxorubicin resistance in HBL-100 cells might not be excluded [40].

ZNF217-mediated paclitaxel resistance in breast cancer cells was associated with alterations in the intrinsic mitochondrial apoptosis pathway, through the deregulation of the balance between anti-apoptotic (Bcl-2 and Bcl-X_L_) and pro-apoptotic (Bad, Bak and Bax) proteins' expression [28]. The *BCL2L1* pre-mRNA gives rise after alternative splicing to the anti-apoptotic Bcl-X_L_ or the pro-apoptotic Bcl-X_S_ transcripts and complex regulation allows the shift of the splicing in favor of one or the other. Aurora-A has been implicated in regulating this splicing [45] and was associated with increased expression of Bcl-X_L_ [46] and resistance to taxol-mediated apoptosis in breast cancer [47]. ZNF217 induced overexpression of both the Bcl-X_L_ and Aurora-A proteins in paclitaxel-resistant ZNF217-overexpressing breast cancer cells [28], and expression of BCL2L1 protein correlated with that of *ZNF217* mRNA in colorectal tumors [48]. The mechanism behind ZNF217-driven paclitaxel resistance thus likely involves, at least in part, Aurora-A overexpression favoring the production of the anti-apoptotic protein Bcl-X_L_.

The anti-apoptotic activity of ZNF217 is also mediated through activation of the PI3K/Akt survival pathway. Indeed, ectopic expression of ZNF217 led to activation of the PI3K/Akt pathway, and silencing *ZNF217* resulted in decreased Akt phosphorylation [19, 40]. A feedback loop seems to exist between ZNF217 and the Akt pathway, as inhibition of PI3K with the LY294002 inhibitor led to decreased ZNF217 protein expression levels and increased apoptosis in response to doxorubicin [40]. ErbB3-driven activation of PI3K/Akt was implicated in resistance to paclitaxel [49] and, to date, the increased expression of the *ErbB3* gene, a direct target for the ZNF217 transcription factor [19], is the only clearly defined mechanism associated with the ZNF217-driven activation of the PI3K/Akt pathway [19, 30]. As Aurora-A [46, 50] and eEF1A2 [36] are also known to activate the PI3K/Akt pathway, one cannot exclude that ZNF217-driven survival and resistance to apoptosis via the PI3K/Akt pathway is also Aurora-A- or eEF1A2-dependent.

### Activating invasion, metastasis and the EMT process

**Figure 2:**
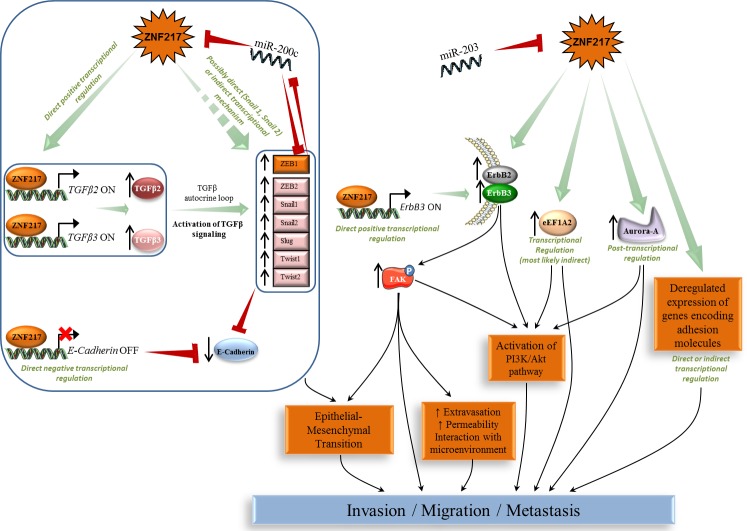
ZNF217-driven orchestration of invasion, metastasis and EMT process Left panel: ZNF217-induced EMT involves the TGF-β pathway and several deregulated expression of EMT key members (E-cadherin, Snail, Slug, Twist and ZEB). Right panel: ZNF217 also induces deregulation in ErbB2-ErbB3, eEF1A2, Aurora-A and several adhesion molecules associated with the activation of complex subcircuits (*e.g.* FAK, PI3K/Akt) leading to EMT, invasion and metastases development.

ZNF217 strongly stimulated migration, invasion and anchorage-independent growth in a soft-agar assay in several *in vitro* breast or ovarian cell models [20, 27, 30, 51]. Conversely, *ZNF217* extinction allowed reversion of these phenotypes in breast, ovarian and colorectal cancer cells [20-22, 29, 52, 53]. miR-203 targeted *ZNF217* mRNA for silencing and was associated with decreased cell migration and invasion in colorectal cancer cells [21]. In mouse MECs, ZNF217 promoted loss of adhesion and increased cell motility associated with reorganization of actin cytoskeleton [30]. ZNF217 xenografts in mice were prone to developing spontaneous metastases, especially in lung, liver and lymph nodes [20, 27, 30] and high *ZNF217* expression levels were associated with metastases in human breast tumors [20]. Several studies have used gene-expression microarrays to extract gene networks in the vicinity of ZNF217 in a cancer context [20, 30, 54, 55] and all revealed by gene ontology an effect on cell adhesion, cell motility, migration and invasion, of ZNF217 deregulated expression. More particularly, identified genes were those encoding proteins involved in cell adhesion, in the focal adhesion pathway or in the TGF-β pathway [20]. The focal adhesion kinase (FAK) signaling [56] was critical for ErbB2/ErbB3 receptor cooperation required for transformation and invasion [57, 58]. In breast cancer cells, ZNF217-induced overexpression of ErbB2 and ErbB3 proteins is paired with increased phospho-FAK levels [20]. eEF1A2 has been implicated in actin remodeling, invasion and migration [36, 59] and silencing *eEF1A2* inhibited the anchorage-independent growth mediated by ZNF217 in ovarian cells [37], again suggesting that eEF1A2 might be a key player in ZNF217-mediated deleterious effects (Figure 2).

Another major finding was that ZNF217 promotes epithelial-mesenchymal transition (EMT) in human MECs, associated with a significant reduction in expression of epithelial markers such as E-cadherin, increased levels of mesenchymal proteins and significantly increased mRNA levels of transcription factors known as major drivers of EMT (*Snail1/2, Twist1/2, ZEB1/2*) [20]. Some of these features were validated in ZNF217-overexpressing mouse MECs [30]. ZNF217 enrichment has been found in the promoters for *Snail1* and *Snail2* genes in human breast cancer cell lines and tumors [13], and the promoter of the *E-cadherin* gene is a direct target for ZNF217 [12]. ZNF217-overexpressing cells also possess sustained activation of the TGF-β pathway as the consequence of a TGF-β autocrine loop, and direct binding of ZNF217 to *TGFB2* or *TGFB3* promoters was found to increase expression and thus secretion of active TGF-βs [20]. Finally, inhibition of the TGF-β pathway led to the reversal of ZNF217-dependent features of EMT and invasion, thus highlighting the TGF-β-activated Smad signaling pathway as a major driver of ZNF217-induced EMT. The authors' proposed model for ZNF217-driven EMT incorporates the direct transcriptional down-regulation of *E-cadherin* expression and/or the constitutive activation of the TGF-β-activated Smad signaling pathway [20]. An increased level of complexity was highlighted with the discovery of miR-200c targeting both *ZEB1* and *ZNF217* mRNAs and of a miR-200c/ZNF217/TGF-β/ZEB1 circuit contributing to EMT and invasion [22]. MiR-200c was shown to target *ZNF217* mRNA, ZNF217 protein to upregulate autocrine TGF-β, and TGF-β signaling to transcriptionally activate ZEB1, which itself could then exert a feedback inhibition on miR-200c [60] (Figure 2). In many late-stage tumors, TGF-β signaling is found redirected from suppressing cell proliferation to EMT activation [61]. ZNF217 thus leads to a compromised TGF-β pathway at later stages of tumorigenesis, via a complex cross-talk that appears to involve, at least, direct transcriptional or miRNA-driven regulating events.

### Cancer stem cells and differentiation

Cancer stem cells (CSCs) possess self-renewal and multilineage differentiation capability and have been proposed as the driving force of tumorigenesis and the seeds of metastases [62]. Recent research has linked the acquisition of CSC traits with the EMT transdifferentiation program [63]. Several investigations revealed that ZNF217 binds to the promoter of genes involved in differentiation and organ development and is associated with deregulated expression of genes involved in repression of cell differentiation and maintenance of CSCs [13, 20, 30]. ZNF217 was also found upregulated in the recently formed somites of mouse embryos compared to presomitic mesoderm [64], supporting a possible involvement in somite development.

Overexpression of ZNF217 in normal primary mammary epithelial cells or breast cancer cells increased the formation of mammospheres displaying self-renewal capacity [20, 30, 51] and was associated with repression of an adult stem cell expression signature that is also found downregulated in cancer [30]. ZNF217-driven increased motility, EMT stimulation and increased clonogenicity are associated with a less differentiated or more mesenchymal phenotype. In normal mammary epithelium, *ZNF217* expression was enriched in the CD24^Med^CD49f^High^ cell population, which includes basal, myoepithelial and progenitor cells, compared with CD24^High^CD49f^Low^ cells, which include luminal and luminal progenitor cells [30]. After transplantation of ZNF217-overexpressing cells in mice, the corresponding tumors presented a more basal pathology with increased dual-positive luminal and basal cell marker expression [30]. Finally, ZNF217 expression was repressed in pluripotent embryonal cells after treatment with the differentiating agent retinoic acid, suggesting that aberrant expression of ZNF217 in differentiated adult cells may suppress differentiation, leading to tumorigenesis [13].

Glioblastoma multiforme is the most aggressive and common type of primary brain tumor that is hypothesized to be driven by from a subpopulation of glioma stem cells (GSCs) [65]. Within these GSCs, ZNF217 is several-fold more highly expressed than in non-GSCs [66]. Forced differentiation of GSCs downregulated ZNF217 and knockdown of *ZNF217* inhibited the proliferation of GSCs and reduced stem-like cell populations [66]. As Aurora-A may regulate self-renewal capacity in glioma-initiating cells via stabilization/activation of β-catenin/Wnt signaling [67], it might also be involved in ZNF217-driven maintenance of a stem-like phenotype. Hypoxia is a tumor micro-environmental factor that is critical for the GSC niche [68]. Importantly, ZNF217 is regulated by hypoxia-inducible factors (HIFs) through direct or indirect mechanisms, suggesting that ZNF217 may be a downstream target of HIFs that promotes the hypoxia-induced stemness of GSCs [66]. In conclusion, ZNF217 plays a role in cell reprogramming by promoting self-renewal, maintaining stem cells and inhibiting differentiation.

### *ZNF217* gene expression product, a powerful biomarker

Amplification at the *ZNF217* locus has been previously reviewed [8] and linked to poor prognosis in several cancers [7, 9-11, 53]; however other authors have failed to make this link [69-72], likely due to differences in probes and cutoff levels used for amplification evaluation. High levels of *ZNF217* expression have been documented in cell lines and tumors in which the locus is amplified [3, 7, 73]; conversely, an absence of correlation between *ZNF217* amplification and *ZNF217* expression levels has also been observed [3, 7, 72-74]. Epigenetic events such as miRNA targeting of *ZNF217* and DNA methylation of *ZNF217* promoter have recently been highlighted providing important insight into the molecular mechanisms regulating ZNF217 expression. Several miRNAs (miR-203, miR-200c, miR-24) targeting the 3′ UTR of *ZNF217* mRNA were found to regulate ZNF217 expression and functions [21-23]. Deregulated methylation status at the *ZNF217* gene promoter has been observed under hypoxia [75] or as part of a relevant DNA methylation pattern detected in sarcoma subtypes [25]. Hypomethylation of the *ZNF217* promoter and *ZNF217* overexpression were inversely correlated (*p* = 6 × 10^−4^) in glioblastoma but not in normal brain tissue [26]. In breast tumors, the methylation status at the *ZNF217* gene promoter correlated with *ZNF217* gene-expression levels [24, 51] while in peripheral blood cells, lack of methylation at the *ZNF217* locus predicted breast cancer risk (*p* = 0.006) [76]. Clearly, investigating the biomarker value of *ZNF217* gene-expression levels, independently of the *ZNF217* amplification status appears worthwhile.

In the original work depicting the *ZNF217* gene, higher *ZNF217* transcript levels were found in breast tumor samples than in the corresponding normal epithelium [3]. Further observations revealed that ZNF217 mRNA and protein were overexpressed in primary prostate carcinoma *versus* normal prostate tissue (*p* < 0.05 and 14 out of 23 analyzed cases, respectively) [23] and in colorectal carcinoma tissues associated with poor clinicopathological features (*p* < 0.05) [52]. High *ZNF217* mRNA levels were present in glioma samples compared to normal tissue and associated with poor outcome (univariate analysis, *p* < 0.001) [66]. RTQ-PCR investigation in several primary breast cancer cohorts found that high *ZNF217* mRNA levels were associated with shorter relapse-free survival (RFS) (univariate analysis, *p* = 0.003, *p* = 0.017 and *p* = 0.02 [20]; *p* = 0.015 [51]) and with the development of metastases (*p* = 0.002, *p* = 0.0008 and *p* = 0.025 [20]). Retrospective analysis of transcriptomic data confirmed that *ZNF217* was a marker of poor prognosis linked with shorter RFS (univariate analysis, *p* = 0.01 [20]; *p* = 0.01 [30]) and overall survival (OS) (univariate analysis, *p* = 0.003 [30]; *p* = 0.037 [51]). The most remarkable results arose from analysis of publicly available microarray data for 2,414 and 2,978 breast cancer patients [77, 78], in which it was found that high levels of expression of *ZNF217* were associated with poor prognosis, with *p* values of, respectively, 1 × 10^−11^ [20] and 3 × 10^−9^ [51]. Interestingly, *ZNF217* mRNA expression was found to be an independent prognostic marker more informative than lymph node (multivariate analysis*, p* < 0.05, [20] or estrogen receptor alpha (ERα) status (multivariate analysis, *p* = 0.04 [30]). In terms of predicting treatment response, retrospective analysis of two independent expression datasets from breast cancer patients who received neoadjuvant chemotherapy, showed lower levels of *ZNF217* mRNA in responders compared to in non-responders (*p* = 0.04 and *p* < 0.0001) [30]. Altogether, these data suggest that in breast cancer, *ZNF217* mRNA expression is both a novel and powerful biomarker for poor prognosis and a prognostic predictor of patient outcome in response to chemotherapy. Investigation of *ZNF217* expression would thus allow the stratification of breast cancer patients into outcome-dependent subclasses.

Recently, immunohistochemistry (IHC) performed on paraffin-embedded tissue samples revealed higher ZNF217 protein expression levels in tumor tissue from both squamous cervical cancer (10 out of 10 tested samples) [79] and colorectal cancer (*p* < 0.05 [52], *p* < 0.001 [21]) compared to in matched normal adjacent tissue. ZNF217 protein levels were associated with aggressive clinical markers (*p* < 0.05 [52] and *p* < 0.05 [21]) and shorter OS (univariate analysis, *p* = 0.028 [21]) in colorectal cancer and with shorter RFS (univariate analysis, *p* = 0.0016) and OS (univariate analysis, *p* = 0.019) in gastric carcinoma [80]. The prognostic value of ZNF217 protein levels in ovarian carcinoma has been found in one study (univariate analysis, *p* = 0.042) [27], but not in two others [7, 72]. The abovementioned studies differed in terms of the anti-ZNF217 antibodies used, the cutoff levels or scores for considering ZNF217 expression signal, as well as the subcellular localization signals taken into account (Table 1). When the information was available, nuclear [7, 72, 80] or cytoplasmic staining only [21] were used. Interestingly, both nuclear and cytoplasmic ZNF217 staining has been used to define an IHC ZNF217 index in a breast cancer study [51]. Nuclear staining corroborated the transcription factor role of ZNF217 and was also observed after ectopic expression of ZNF217 in several cell lines [73]. No cytoplasmic ZNF217 function has yet been deciphered, though several lines of evidence indicate that it can localize in both the nucleus and the cytoplasm. In OSE cells transduced with *ZNF217*, the ZNF217 protein was prominent in the nuclei of stationary senescent cells and cytoplasmic in proliferating cells [35], suggesting that ZNF217 localization may depend on the cells' proliferative state. More recently, subcellular fractionation of MCF-7 breast cancer cells revealed the presence of endogenous ZNF217 protein predominantly in the nuclear fractions, but also in the cytoplasmic fraction [51]. Surprisingly, although ZNF217 cytoplasmic staining was visible in some IHC pictures, it was neither mentioned nor discussed by the authors (Table 1). These new findings support the existence of a ZNF217 protein pool of unknown biological function, localized in the cytoplasm of cancer cells. Altogether, evaluating ZNF217 protein biomarker value in tumor samples is complex, and the expected data from future studies dedicated to deciphering the meaning of ZNF217 cytoplasmic localization may help in considering ZNF217 nuclear and/or cytoplasmic signals in translational medicine.

**Table 1:** Immunohistochemistry (IHC) investigation of ZNF217 expression in normal and tumor tissue samples

Type of cancer	Number of biological samples tested	Primary antibody used for ZNF217 detection		Subcellular localization signal considered by the authors	Scoring	Cutoff level for ZNF217 positivity	% of ZNF217-positive samples	Analyses and observations	Personal comments	Ref.
		Species	Suppliers (References)							
Squamous cervical cancer (SCC) and matching adjacent normal cervical tissues	10 pairs	Rabbit (polyclonal)	Santa Cruz (clone not specified)	Cytoplasmic and diffuse	Not described	Not described	All the tested SCC tissues were positive for ZNF217; while normal cervical cells were weakly positive for ZNF217	Overexpression of ZNF217 protein in SCC compared to normal cervical tissue. Validated by Western Blot (p < 0.01)		[79]
Colorectal carcinoma (CRC) and matching non tumor adjacent tissues	60	Rabbit (polyclonal)	Santa Cruz (sc-67223)	Not described by the authors	Calculated by multiplying the staining intensity and the percentage of positive cells	Not described	81%	Significant ZNF217 overexpression in CRC versus non tumor adjacent tissues (*p* < 0.05)	Presence of a clear cytoplasmic staining, but a nuclear staining may also be present in the article's pictures	[52]
Gastric carcinoma	84	Goat (polyclonal)	Abcam ab136678)	Nuclear	0, no detectable signal 1+, weak staining 2+, moderate staining 3+, strong staining	2+ and 3+	40.5%	Poor prognostic factor associated with shorter RFS (*p* = 0.0016) and OS (*p* = 0.019); independent prognostic factor for RFS (*p* = 0.02)		[80]
Ovarian clear cell carcinoma (OCCC)	68	Rabbit (polyclonal)	Sigma Aldrich (clone HPA051857)	Nuclear	0, undetectable signal 1+, weakly positive 2+, moderately positive 3+, intensively positive	2+ and 3+	40%	No association with PFS or OS	The nuclear staining was taken into account for IHC, but a cytoplasmic staining was clearly visible in the article's pictures	[72]
OCCC	60	Rabbit (polyclonal)	Abcam (clone not specified)	Nuclear	0, undetectable signal 1+, weakly positive 2+, moderately positive 3+, intensively positive	2+ and 3+	33%	No association with PFS	The nuclear staining was taken into account for IHC, but a cytoplasmic staining was clearly visible in the article's pictures	[7]
Ovarian tumors	44	Not described for the IHC investigation		Not described by the authors	Staining score (from 0 to 7) is the sum of staining intensity (0, negative; 1, weak; 2, medium; 3, strong) and % of positive stained cells (0, 0%; 1, 1-25%; 2, 26-50%; 3, 51-75%; 4, 76-100%)	Staining score ≥ 3	59% (highly expressed in 35%)	Poor prognostic factor associated with shorter DFS (*p* = 0.042)		[27]
Colorectal tumors	82	Rabbit (polyclonal)	Biosynthesis Biotechnology (clone not specified)	Cytoplasmic	The cutoff score is the closest score with both maximum sensitivity and specificity according to receiver operating characteristic (ROC) curves	Staining score above the cutoff score	76.3%	Poor prognostic factor associated with shorter OS (*p* = 0.028); ZNF217 expression positively correlated with tumor size, depth of invasion and lymph node (*p* < 0.05)		[21]
Primary breast tumors	162	Rabbit (polyclonal)	Abcam (ab48133)	Nuclear and cytoplasmic	Nuclear and cytoplasmic staining was used to define an IHC ZNF217 index	High IHC ZNF217 index: high % of tumor cells displaying nuclear staining (> 60%) and a high % of cells displaying cytoplasmic staining (> 70%)	60% displayed both nuclear and cytoplasmic staining, 27% displayed a nuclear staining only, 9% displayed cytoplasmic staining only and no staining could be detected in 4% of the samples	High ZNF217 index is a poor prognostic marker associated with shorter RFS in luminal-A (*p* = 0.01), but not in luminal-B breast cancers; Predictive value for endocrine therapy only (*p* = 0.05)	Validation of both nuclear and cytoplasmic ZNF217 localization after subcellular fractionation of MCF-7 cells by Western Blot and by PLA (Proximity Ligation Assay)	[51]

RFS, relapse-free survival; OS, overall survival; PFS, progression-free survival; DFS, disease-free survival.

### ZNF217 specific role and biomarker value in ERα-positive breast cancers

Compelling data indicate a specific behavior of ZNF217 in an ER+ context of breast cancers and a close interplay between ZNF217 and ERα signaling. The first evidence of this came from the observation that *ZNF217* is (directly or indirectly) an ERα target gene, as 17β-estradiol exposure led to *ZNF217* mRNA deregulated expression in ER+ breast cancer cells [81]. Further investigations revealed that estrogen signaling disruption, such as siRNA targeting ERα, could trigger epigenetic silencing (hypermethylation) of the *ZNF217* gene and that the methylation status at the *ZNF217* locus was higher in ERα-negative (ER-) *versus* ER+ breast tumors [24]. In peripheral blood cells, lack of methylation at the *ZNF217* locus, predicting breast cancer risk, was associated with ERα bioactivity in the corresponding serum [76]. ER+ breast tumors or cancer cell lines displayed higher ZNF217 expression levels compared to their ER- counterparts (*p* = 0.0004, *p* = 0.005 and *p* = 1 × 10^−7^ [51]; *p* = 0.009 and *p* = 0.0001 [82]). In an ER+ breast cancer context, high/positive ZNF217 expression levels thus seem to be associated with functional estrogen signaling.

**Figure 3:**
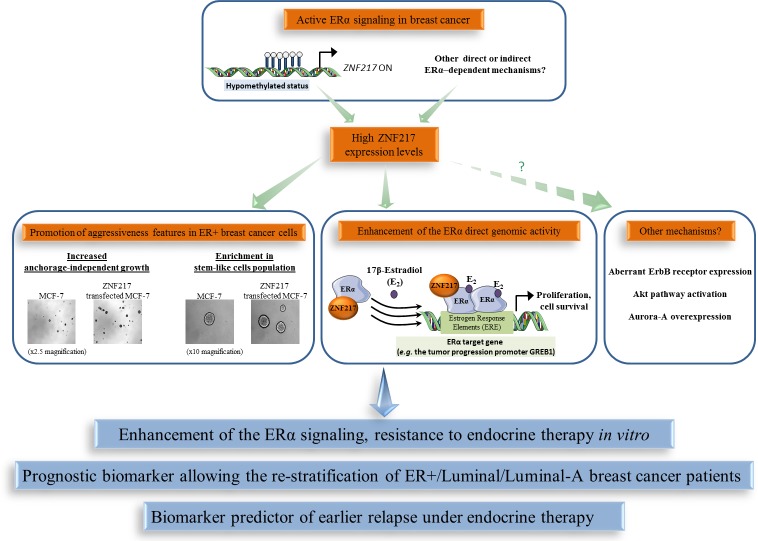
The functional interplay between ZNF217 and ERα in breast cancer High ZNF217 expression levels correlates with an active ERα signaling context in breast cancer, enhancement of ERα signaling and endocrine therapy resistance. ZNF217 is also a new powerful prognostic or predictive biomarker for endocrine therapy in breast cancer.

Besides the link between ZNF217 expression and ERα status, a physical interaction between ERα and ZNF217 proteins has been reported [51, 82, 83]. Indeed, ZNF217 and ERα proteins interact via the C-terminus region of ZNF217 and the hinge region of ERα, and the interaction was visible both in the nucleus and in the cytoplasm [51]. ZNF217 is capable of enhancing ERα ligand-dependent classical genomic activity, at least in part, by increasing the recruitment of ERα to its estrogen response elements (demonstrated at the endogenous level for the *GREB1* gene, encoding for a growth promoter protein) [51]. The significance of the cytoplasmic interaction between ZNF217 and ERα is currently unknown, but one hypothesis is that ZNF217 also modulates the non-genomic estrogen signaling, which is linked to the activation of the Akt signaling cascade [84]. A model for ZNF217-driven deleterious functions in ER+ breast cancer cells involving interference with and enhancement of ERα signaling and its related mitogenic downstream events, at least via the direct genomic activity of ERα, is proposed (Figure 3). A second study dedicated to ZNF217 chromatin occupancy in the ER+ MCF-7 breast cancer cell line supported these findings, revealing that ZNF217 binding sites overlap with those of transcription factors belonging to the ERα network (ERα, GATA3 and FOXA1) [82].

Assessing the prognostic value of *ZNF217* mRNA expression levels (RTQ-PCR) in the molecular classification of breast cancers, revealed it as more informative in luminal breast cancers (*p* = 0.006, univariate analysis for RFS; *p*= 0.028, univariate analysis for OS) than in HER2+ or triple negative subtypes (no significance) [51]. The luminal-A subtype is considered to have the most favorable prognosis, exhibiting higher expression levels of *ESR1*, *ERα* and *ER*-related genes and a lower proliferative index than the luminal-B subtype. Of utmost interest, two studies revealed that the prognostic power of both *ZNF217* mRNA (*p* = 0.02, considering RFS, [51]; *p* = 0.035, considering OS, [82]) and ZNF217 protein (*p* = 0.01 for RFS, [51]) (IHC ZNF217 index, Table 1) expression levels were most discriminatory in the luminal-A subtype compared with the luminal-B subtype [51, 82]. Retrospective analysis of gene-expression array data for 2,978 breast cancer patients revealed that high levels of *ZNF217* mRNA expression were strongly and significantly associated with shorter RFS in the luminal subgroup (*p* = 2.2 × 10^−5^), but were not prognostic in HER2+ or triple negative subclasses. Again, the most powerful prognostic value of *ZNF217* was observed in the luminal-A subgroup (*p* = 3.3 × 10^−4^), compared with the luminal-B subclass (*p* = 0.07).

ZNF217 has also been associated with aberrant response of endocrine therapy (a standard of care for ER+ breast cancers). *ZNF217* expression was deregulated in endocrine therapy-resistant breast cancer cells [85]. Ectopic expression of ZNF217 conferred aggressiveness and tamoxifen endocrine therapy resistance to ER+ breast cancer cells, while *ZNF217* silencing restored tamoxifen sensitivity in resistant breast cancer cells [51]. Finally, high ZNF217 mRNA (univariate analysis, *p* = 0.02, *p* = 0.011, *p* = 0.047, *p* = 0.035) or protein levels (*p* = 0.05) in human ER+ primary breast cancer tumors were predictors of earlier relapse under endocrine therapy only (Table 1) [51]. This was further validated by another group at the *ZNF217* mRNA level (*p* = 0.05) [82]. Further work is however necessary in the neoadjuvant setting to confirm the predictive value of ZNF217 for endocrine therapy response.

Altogether, these data support the idea that the functional interplay existing between ZNF217 and ERα could be responsible, in tumors expressing high levels of ZNF217, for an altered response to endocrine therapy and poor outcome (Figure 3). However, other ZNF217-driven mechanisms such as aberrant ErbB receptor expression, Akt pathway activation, Aurora-A expression, enrichment in stem-like cell population, all already linked with endocrine therapy resistance [86-88], could also be involved. In conclusion, the prognostic value of ZNF217 appears most powerful in the breast tumor subtypes where ERα expression and ERα signaling are more prominent, which may reflect the impact of ZNF217 in modulating existing active ERα signaling. ZNF217 expression levels thus allow the re-stratification of breast cancer patients considered as having a good prognosis, for whom no other widely used biomarker is currently available. Assessing ZNF217 expression levels may thus help distinguish patients with an excellent outcome who would benefit from endocrine therapy only (possessing low ZNF217 expression levels), from those harboring tumors possessing high ZNF217 expression levels that might thus poorly respond to endocrine therapy and would need more aggressive therapy (endocrine therapy combined to chemotherapy).

### Implications of experimental findings for clinical medicine

Using *in silico* and *in vitro* screening approaches to identify candidate therapeutics able to inhibit growth of cancer cells expressing high ZNF217, triciribine (also known as API-2) was revealed as a good drug candidate [30]. This nucleoside analogue inhibits DNA synthesis and allosterically inhibits AKT activation, and has been tested in phase I clinical trials in patients with cancer as well as in one phase II clinical trial in patients with metastatic breast cancer [89-91]. Triciribine overcomes *in vitro* ZNF217-induced doxorubicin resistance in breast cancer cells, and in mice experiments is more effective in inhibiting tumor growth of ZNF217-overexpressing cells than control tumors [30]. To date, it is the only drug tested *in vivo* that seems to counteract the ZNF217-driven deleterious effects and has been proposed as a clinical strategy to treat ZNF217+ cancer patients.

Among the alternative candidate strategies, miRNAs represent a class of promising targets for therapeutic intervention [92]. In particular, miR-200c, which targets both *ZNF217* and *ZEB1,* re-sensitized trastuzumab-resistant breast cancer cells to trastuzumab *in vitro* and suppressed metastases *in vivo* [22]. The Aurora-A kinase inhibitor III was efficient in reversing paclitaxel resistance in ZNF217-overexpressing breast cancer cells *in vitro* [28]. ZNF217-mediated EMT in normal mammary epithelial cells could be reversed by treatment with SB431542, which is an ATP analog and inhibitor of the kinase activity of the TGF-β type I receptor, or by a siRNA-based strategy targeting Smad4 [20]. Altogether, clinical strategies counteracting ZNF217-mediated effects, either directly (miRNA or siRNA *ZNF217* silencing) or by targeting its possible key-mediators (Aurora-A, FAK, PI3K/Akt and TGF-β signaling pathways) would represent valuable alternative approaches for the management of the subpopulation of ZNF217+ breast tumors possessing poor prognosis.

## CONCLUDING REMARKS AND FUTURE DIRECTIONS

**Figure 4:**
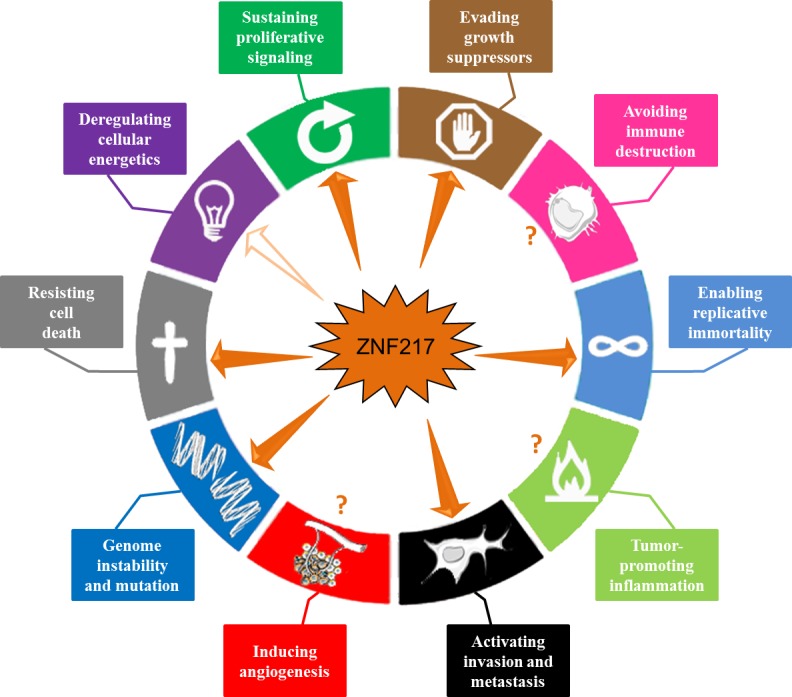
ZNF217 interferes with the major hallmarks of cancer ZNF217 governs or interferes with several hallmark capabilities within cancer cells: genome instability and mutation, sustained proliferative signals, growth suppressor evasion, replicative immortality, resistance to cell death and invasion activation (depicted with plain arrows). Recent data suggest that ZNF217 might play a role in reprogramming energy metabolism (indicated by an empty arrow). ZNF217 interplay with immune destruction escape, inflammation or angiogenesis deregulation remains unknown (depicted with question marks).

In conclusion, accumulating data indicate that ZNF217 is a key player in tumorigenesis, orchestrating tumor progression at both early and late stages. ZNF217 cooperates with several intracellular signaling networks to reprogram integrated circuits governing hallmark capabilities within cancer cells. In this review, we have extensively documented the implication of ZNF217 in the major hallmarks of cancer, including sustained proliferative signals, growth suppressor evasion, replicative immortality, resistance to cell death and invasion activation (Figure 4). The direct or indirect downstream actors are Aurora-A, eEF1A2, ErbB2/ErbB3, p15^ink4b^, members of the Bcl-2 family, E-cadherin and ERα, and are all involved in at least one, but most of the time in several of the above-cited cancer features. ZNF217-driven molecular functions are multiple and involve ZNF217 direct binding at the promoter of target genes (*ErbB3, E-cadherin, TGFB2, TGFB3*), indirect transcriptional regulation (*eEF1A2*), epigenetic regulation (*p15^ink4b^*), post-transcriptional events (Aurora-A) and protein-protein interaction (ERα). Evidence of a close interplay between ZNF217, or its molecular mediators, and the TGF-β or the PI3K/Akt signaling pathways has emerged recurrently, pointing out the importance of these subcircuits and adding to the complexity with regards the considerable interconnections existing between these individual mediators.

The *ZNF217* gene belongs to the 20q13 region, a region frequently amplified in human cancers and importantly, ZNF217 protein interferes and cooperates with proteins encoded by other genes present in this region (*AURKA, BCL2L1, eEF1A2*). Interestingly, the prognostic significance of *ZNF217* amplification seems to be variable and depend on the concomitant presence of co-amplified loci at 20q13 [74]. A model can be proposed whereby ZNF217 cooperates with Aurora-A, BCL2L1 or eEF1A2 in neoplastic progression of breast cancer through genomic co-amplification and/or through ZNF217-driven molecular regulation, which might amplify the impact of any genomic amplification.

In many human tumors, the pervasive genomic aberrations at the *ZNF217* locus have provided clear evidence for loss of control of genome integrity [16] (Figure 4). Genome instability not only appears to be associated with destabilization of gene copy number, but also with nucleotide sequence polymorphisms or DNA repair defects. The rs1056948 single nucleotide polymorphisms (SNP) in the 3′ UTR of *ZNF217* (with a putative function in inactivating exonic splicing enhancer sequences) and the rs61730988 SNP (responsible for the E914D mutation) are associated with breast cancer susceptibility [93, 94]. While ZNF217 has not yet been linked to impaired DNA repair, it has been described as a direct repressor of *BRCA1*, through cooperation with a histone demethylase [17]. Further studies are expected to unravel the possible role of ZNF217 in DNA repair defect or whether *ZNF217* polymorphisms belong to the roster of mutant genes needed to orchestrate tumorigenesis.

Whether ZNF217 interplays with immune destruction escape, inflammation, angiogenesis or cellular energetics deregulation remains unknown (Figure 4). Transcriptomic analyses have however revealed that deregulated ZNF217 expression is paired with aberrant expression of enzymes involved in metabolism [20]and that ZNF217 might interfere with lipid metabolism [54]. More precisely, ZNF217 might interfere with the lysophosphatidylcholine acyltransferase 1 [54] recently implicated in breast cancer progression and metastatic dissemination [95]. Future studies should provide new exciting data concerning the role of ZNF217 in reprogramming energy metabolism and in producing oncometabolites.

## Abbreviations

CSCs: cancer stem cells
DFS: disease-free survival
EMT: epithelial-mesenchymal transition
ERα: estrogen receptor alpha
ER+: ERα-positive
ER-: ERα-negative
FAK: focal adhesion kinase
GSCs: glioma stem cells
HIFs: Hypoxia-inducible factors
HMECs: human mammary epithelial cells
IHC: immunohistochemistry
MECs: mammary epithelial cells
OS: overall survival
OSE cells: ovarian surface epithelial cells
PFS: progression-free survival
RFS: relapse-free survival
TRF2: telomere repeat binding factor 2
ZNF217: zinc-finger protein 217
